# Assessment of the effects of sex, age, and rearing condition on ultrasonic vocalizations elicited by pups during the maternal potentiation paradigm in C57BL/6J mice

**DOI:** 10.1002/dev.22341

**Published:** 2022-11-12

**Authors:** Danielle Santana‐Coelho, Paige D. Womble, Katherine J. Blandin, Jacob B. Pilcher, Grace M. O'Neill, Leighton A. Douglas, Srikhar V. Chilukuri, Doan L. K. Tran, Taylor A. Wiley, Joaquin N. Lugo

**Affiliations:** ^1^ Department of Psychology and Neuroscience Baylor University Waco Texas USA

**Keywords:** maternal potentiation, mice, rearing, repertoire, ultrasonic vocalizations

## Abstract

Isolation‐induced ultrasonic vocalizations (USVs) are important to elicit parental retrieval. This behavior is critical for the animal's survival and can be altered in models of developmental disorders. The potentiation of vocalizations in response to reunion with the dam, also called maternal potentiation, has been extensively studied in rats. However, the assessment of this paradigm in mice is scarce. In rats, the potentiation of vocalizations is dependent on rearing conditions. Since mice are the main species used for genetic models of diseases, we aimed to investigate how different factors such as age, sex, and rearing conditions can affect the potentiation of vocalizations in the maternal potentiation paradigm in mice. We carried out experiments using biparental (dam and sire) or uniparental rearing (dam). Pups were tested on postnatal days (PD) 9 or 12. Pups showed increased potentiation in both sexes at PD9 with uniparental rearing. Both rearing conditions and ages changed the repertoire from the first to the second isolation. Spectral parameters were affected by sex, rearing condition and reunion at PD9. At PD12, only duration was altered by reunion. We conclude that the performance of the pups in the maternal potentiation paradigm is dependent on age, sex, and rearing condition.

## 1 INTRODUCTION

Vocal communication is paramount for the survival of many species. Primates and other animals use vocalizations to navigate courtship, locate food sources, and communicate the presence of predators (Bastos et al., 2018; Caruso et al., 2020). Thus, several behavioral tests have been developed to identify the neural substrates responsible for communication in different social situations and assess deficits in communication (Caruso et al., 2020; Shair et al., 2012; Muller & Shair, 2016; Tschida et al., 2019). A commonly used paradigm to identify early communication deficits in rodents is the isolation‐induced ultrasonic vocalization (USV) test (Malkova et al., 2012a; Scattoni et al., 2009). In this test, pups are isolated from the dams and the litter, and this isolation elicits USVs in pups during the first two postnatal weeks.

Animal models of developmental disorders with communication deficits, such as autism, show alteration in the number of vocalizations performed by pups when separated from the dams and litter (Scattoni et al., 2009; Young et al., 2010). Vocalization development through time and other parameters, such as the type of call emitted, is also altered in different disease models (Binder & Lugo, 2017; Malkova et al., 2012a; Young et al., 2010). Although this behavior paradigm has been extensively used to assess communication deficits in early life, additional layers of the complex interactions between the caregivers (dam and sire) and the offspring are not possible to be evaluated using this behavior test.

A serendipitous finding by Hofer and colleagues identified that a brief reunion between an isolated rat pup and the dam induce an increase in the number of vocalizations performed by a pup during a second isolation period (Hofer et al., 1993). This finding led to the development of the maternal potentiation paradigm that is used to assess an additional layer of communication thought to be underscored by filial attachment. Most infant mammals vocalize when isolated and rodents are no different. When a pup is isolated it vocalizes. By being retrieved, the pup connects the vocalization to the retrieval and adapts its behavior by vocalizing more in the next isolation. This adaptive response is dependent on recognizing cues from the dam and is thought to reflect the attachment of the pup to its caretaker. The potentiation is also interpreted as the output of the learning component of the paradigm where the pup learns by association that the vocalization elicits retrieval (Shair, 2014). The maternal potentiation paradigm was developed in rats where the reunion with the dam, sire, and/or the litter can lead to a potentiation of vocalizations (Brunelli et al., 1998; Hofer et al., 1993, 1998). However, the literature using this paradigm in mice is scarce. Since most of the genetic models of developmental disorders are done in mice, it is important to further characterize the effect of different variables in the potentiation of vocalizations in this paradigm in mice.

An early study exploring the factors impacting the potentiation of USVs in rat pups has shown that USV call numbers can be influenced by rearing conditions (Brunelli et al., 1998). In this study, Brunelli and collaborators showed that potentiation of USVs is higher in pups reared in a uniparental than in pups reared in a biparental condition. Rearing conditions, such as biparental vs uniparental rearing, can affect development leading to anxiety‐like behavior and aggressivity (Marler et al., 2008; Yohn et al., 2018). However, the effect of rearing conditions on the potentiation of USVs has not been investigated in mice. Other factors that have not been investigated in this paradigm are the effects of sex differences on potentiation and the age when the pups are tested influence the outcome. Thus, in the present study, we aimed to do a systematic investigation to identify if rearing conditions can influence potentiation in mice pups by assessing the effects of the presence and absence of the sire during rearing. C57BL/6J mice pups, a strain commonly used in research laboratories, of different ages and sex were used in the study to investigate the effect of rearing, sex, and age in the maternal potentiation paradigm.

## 2 METHODS

### 2.1 Mice

Male and female C57BL/6J mice were bred and group‐housed at Baylor University housing facility in standard laboratory conditions. Room temperature was maintained around 22°C with a 12‐h light/dark cycle, and food and water were provided *ad* *libitum*. A maximum of two pups per sex per litter were used to avoid litter effects. Procedures were conducted according to Baylor Institutional Animal Care and Use Committee and the National Institute of Health Guidelines for Care and Use of Laboratory Mice.

### 2.2 Behavior

**FIGURE 1:**
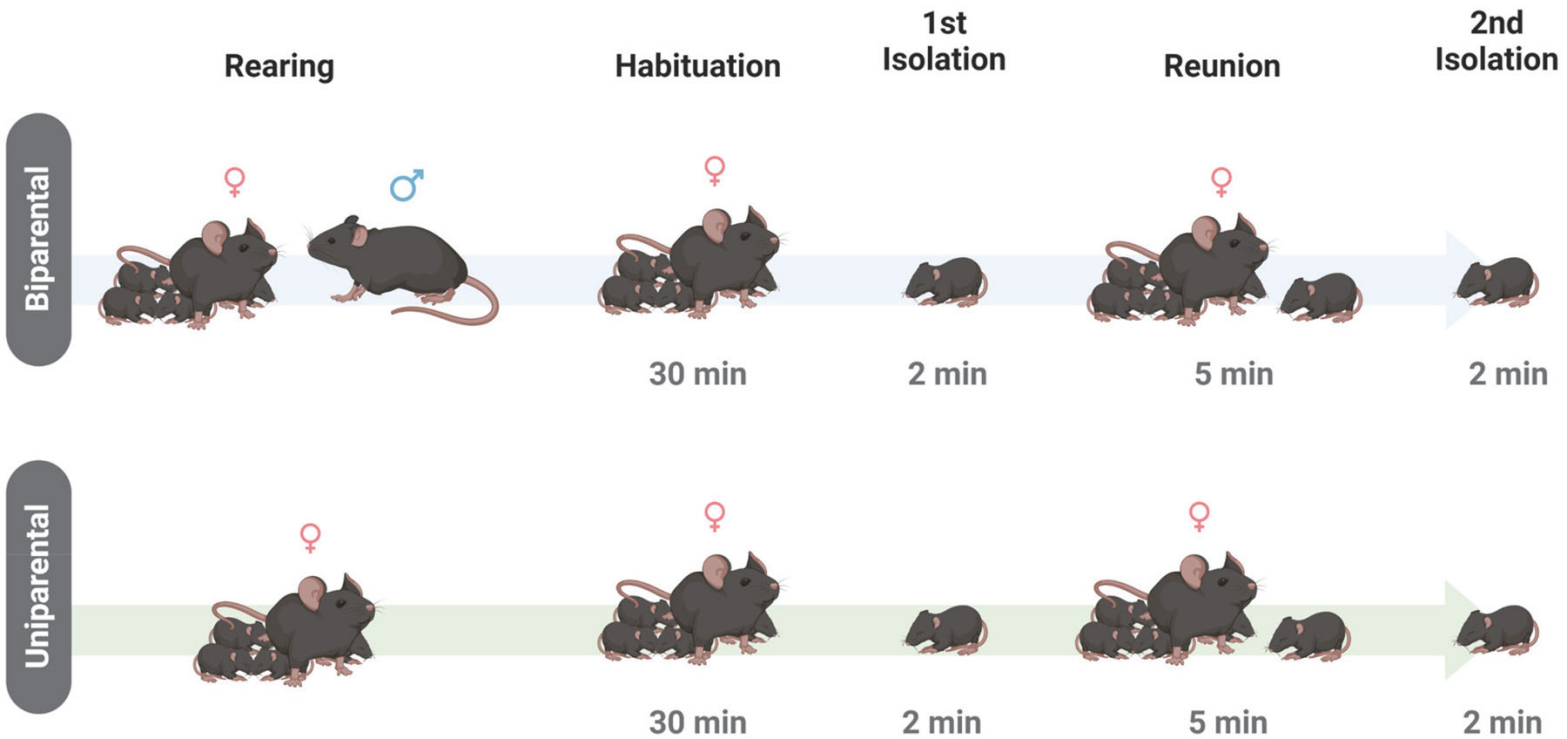
**Experimental design**. *Protocol 1—Biparental Rearing*. Pups were reared by the dam and the sire. On the testing day, the offspring were habituated to the testing room in the home cage with the dam for 30 min. After habituation, one pup was isolated in the recording chamber for 2 min and then reintroduced into the home cage with the dam and the litter. After retrieval by the dam, reunion with the dam and the litter occurred for 5 min. The pup was then isolated again and recorded for 2 min. *Protocol 2—Uniparental Rearing*. Pups were reared only by the dam. Testing procedures occurred the same as in Protocol 1

Mice were weighed and marked prior to testing. To investigate the effects of rearing in the maternal potentiation paradigm, we used two protocols. In Protocol 1, the dam and the sire were in the home cage with the pups during rearing. Before testing started, the sire was taken out of the cage and only the dam and litter were habituated in the behavior room for 30 min. In Protocol 2, once the dam's pregnancy was identified, the sire was separated into a different cage. In this protocol, the litter never had any contact with the sire. On the testing day, the litter and the dam were habituated in the testing room for 30 min (Figure 1).

After habituation, each pup was placed in a plastic container (6 ¼ × 4 ¼ × 5 ⅝ inches) with clean bedding located inside a plexiglass sound‐attenuating chamber. Baseline vocalizations (first isolation) were recorded for 2 min. The pup was reintroduced into the home cage and placed in the corner farthest from the litter and dam. Latency for retrieval was recorded. Reunion time started when the dam retrieved the isolated pup or when the pup crawled to reunite with the litter. After 5 min of reunion, pups were isolated for the second time, and USVs were recorded for 2 min (Figure 1). The procedure was repeated until two pups per sex were tested from each litter. Maternal potentiation of USVs was tested at postnatal days (PD) 9 or 12 and different pups were used for each timepoint. All recordings were done during the light cycle between 12 and 4 pm. A total of 74 animals were used in the study. Thirty‐eight pups were used for Protocol 1 (PD9 females *n* = 10, PD9 males *n* = 10, PD12 females *n* = 9, and PD12 males *n* = 9). Thirty‐seven pups were used for Protocol 2 (PD9 females *n* = 9, PD9 males *n* = 9, PD12 females *n* = 9, and PD12 males *n* = 10). Each group in the study represents five different litters, with a maximum of two pups by sex per litter to avoid litter effects.

### 2.3 USVs recording analysis

Vocalization recordings were done using a condenser microphone (CM16/CMPA, Avisoft Bioacoustics, Germany) connected to an ultrasound‐recording interface (UltraSoundGate 116Hb, Avisoft Bioacoustics). Calls counting was done using DeepSqueak (Coffey et al., 2019) on MATLAB 2018b. A short rat call network was used for the detection of USVs in the recordings with the detection settings set as “high recall” to ensure the detection of as many calls as possible. The other detection parameters were set as followed: total analysis length of 0, analysis chunk length of 6, frame overlap of 0.001 s, frequency low cut‐off of 30 kHz, frequency high cut‐off of 120 kHz, and score threshold of 0. After detection was finalized, the files were revised by an experimenter blind to testing conditions to exclude false‐positive findings. Vocalizations were manually classified as previously described (Romano et al., 2013). Calls were classified into a syllable repertoire of 11 different syllables: chevron, complex, composite, downward, flat, frequency step, short, two‐component, upward, unstructured, and calls that did not fit any of the syllables classifications were called unknown. Due to the low number of unknown calls, this type of USV was not included in the repertoire analysis.

### 2.4 Statistical analysis

Statistical analysis was done using GraphPad Prism 7 software (La Jolla, CA, USA) and IBM SPSS Statistics 28 (Armonk, NY, USA). Data are represented as mean ± standard error mean (SEM). Repeated‐measures three‐way analysis of variance (ANOVA) was used with within subjects factor of “reunion” (before and after) and between‐subject factors of “rearing” (biparental and uniparental) and “sex” (males and females). Two‐way ANOVA was used to analyze the interaction between rearing and sex in retrieval time. Data that presented a significant interaction were further analyzed with Bonferroni's *post hoc* to determine the significance of the differences between the means of the different groups. Pearson correlation was done to assess the relationship between USVs potentiation (change from baseline) and retrieval time. Percentage change between the number of vocalizations performed in the first versus the second isolation was calculated as [(number of USVs emitted in the 2nd isolation × 100)/ number of USVs emitted in the 1st isolation] − 100. Statistical significance was considered when *p* < .05.

## 3 RESULTS

### 3.1 Maternal potentiation is dependent on age and influenced by rearing condition

**FIGURE 2:**
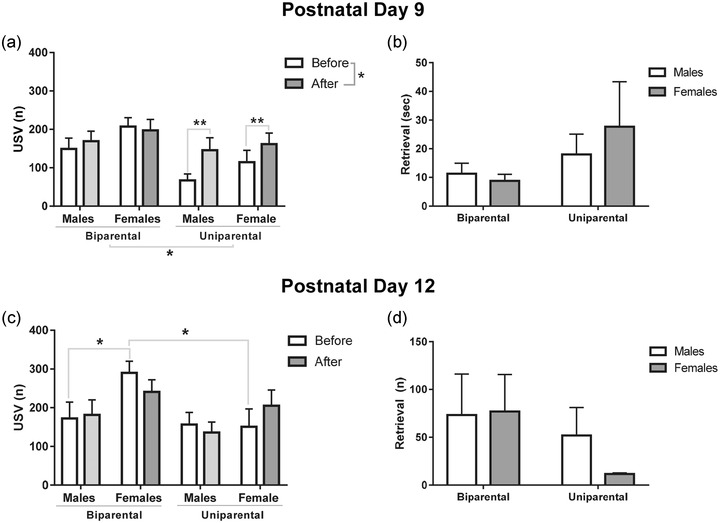
**Maternal potentiation is dependent on age and influenced by rearing conditions**. (a) Total number of calls performed by female and male pups before and after the reunion at PD9 in the biparental and uniparental protocol. The number of calls increased after reunion and the total number of USVs performed by the biparental reared pup was higher than the uniparental reared pups. (b) Latency to retrieval during a reunion at PD9 shows no effect of rearing condition or sex. (c) Total number of calls at baseline (“before”) performed by females pups reared in the biparental protocol was higher than males in the same rearing condition and females in the uniparental rearing. (d) Latency to retrieval during a reunion at PD12 demonstrated no effect of rearing condition or sex. Data are expressed as mean ± SEM. Total number of calls was analyzed with a three‐way repeated‐measures ANOVA with Bonferroni post‐hoc. Retrieval time was analyzed with a two‐way repeated‐measures ANOVA. *N* = 9–10. * *p* < .05 and ** *p* < .01

A three‐way repeated measures ANOVA revealed a within‐subjects main effect of reunion demonstrating a potentiation of vocalizations from before to after reunion (*F*_1,34_ = 5.56, *p* = .02). No interaction between reunion and sex (*F*_1,34_ = 1.13, *p* = .29), or reunion, sex, and rearing conditions (*F*_1,34_ = 0.001, *p* = .970) reached statistical significance (*p* < .05). However, there was a trend for an interaction between reunion and rearing conditions (*F*_1,34_ = 3.99, *p* = .05). A further analysis with a Bonferroni *post hoc* revealed that there was a significant difference in the number of USVs before and after the reunion in the uniparental rearing protocol (*p* < .01). There was no difference between before and after the reunion in the biparental rearing condition (*p* = .79), suggesting that the main effect of reunion is caused mainly by the potentiation in the uniparental rearing protocol. Between‐subjects analysis showed a main effect of rearing (*F*_1,34_ = 6.32, *p* = .01), indicating that pups reared only by the dam (uniparental rearing) vocalize less than pups reared by the dam and the sire (biparental rearing). No main effect of sex (*F*_1,34_ = 2.56, *p* = 1.11) or interaction between rearing and sex (*F*_1,34_ = 0.06, *p* = .80) was identified (Figure 2a). To investigate if retrieval time could potentially account for any differences we see in potentiation, we assessed how long it took for the pups to be retrieved during reunion. At PD9, there was no interaction of rearing and sex (*F*1,34 = 0.51, *p* = .47), and no main effect of rearing (*F*1,34 = 2.28, *p* = .13), or sex (*F*1,34 = 0.17, *p* = .67; Figure 2b).

At PD12, a within‐subjects analysis revealed no significant main effect of reunion (*F*_1,33_ = 0.10, *p* = .92). Also, no interactions were identified between reunion and rearing (*F*_1,33_ = 1.24, *p* = .27), and reunion and sex (*F*_1,33_ = 0.66, *p* = .79). There was a trend for an interaction between reunion, rearing, and sex (*F*_1,33_ = 4.12, *p* = .050). Further analysis with a Bonferroni *post hoc* revealed that female baseline USVs (“before”) in the biparental rearing protocol are higher than males’ baseline (*p* = .03) in the same rearing protocol, and higher than baseline USVs of females in the uniparental rearing protocol (*p* = .01). However, the baseline USV number performed by females in the uniparental rearing protocol does not differ from the number of USVs performed by males reared in the same protocol (*p* = .91; Figure 2c). Between subjects analysis revealed no main effect of rearing (*F*_1,33_ = 3.34, *p* = .07) or sex (*F*_1,33_ = 3.46, *p* = .07). Additionally, no interaction between rearing and sex was identified (*F*_1,33_ = 0.77, *p* = .38). Analysis of retrieval time showed no interaction between rearing and sex (*F*_1,34_ = 0.45, *p* = .50), main effect of rearing (*F*_1,34_ = 1.75, *p* = .19), or main effect of sex (*F*_1,34_ = 0.31, *p* = .57, Figure 2d).

To further assess if potentiation was dependent on the time that the dams took to retrieve the pups, we performed a Pearson correlation between percentage change in the number of USVs from baseline versus retrieval time. No significant correlation was identified when analyzing the number of calls performed by females, males, or both sexes collapsed on PD9 and PD12 (Table 1).

**TABLE 1:** Correlation between retrieval time and percentage change of vocalizations from baseline

Protocol	Postnatal day	Sex	*R* ^2^	*p* value
Protocol 1	PD9	Female	0.0004	.9550
		Male	0.0076	.8096
		Both	0.0014	.8722
	PD12	Female	0.2590	.1618
		Male	0.3895	.0724
		Both	0.1573	.1032
Protocol 2	PD9	Female	0.0008	.9418
		Male	0.0486	.5684
		Both	0.0330	.4704
	PD12	Female	0.0915	.4287
		Male	0.0498	.5351
		Both	0.0030	.8223

To assess if potentiation was dependent on the time that the dams took to retrieve the pups, we performed a Pearson correlation between percentage change in the number of USVs from baseline vs retrieval time.

### 3.2 Reunion alters syllable repertoire in both rearing conditions and ages

**FIGURE 3:**
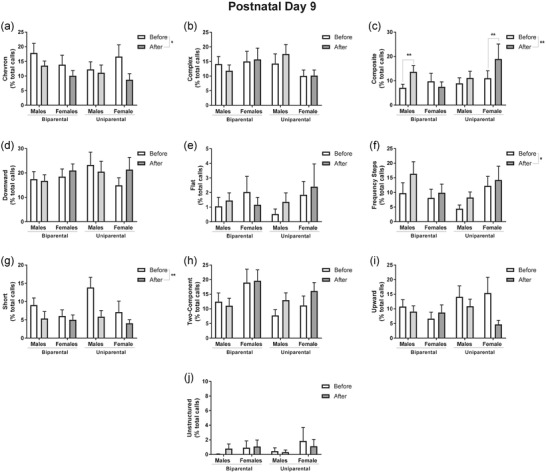
**Reunion alters repertoire at PD9**. Graphs represent the different types of calls performed by the pups during isolation before and after reunion. Each call is represented as the % of the total number of calls in the repertoire. Reunion significantly altered the calls chevron (a), composite (c), frequency steps (f), and short (g). The calls complex (b), downward (d), flat (d), two components (h), upward (i), and unstructured (j) were not altered by reunion. Data are expressed as mean ± SEM and analyzed with three‐way repeated‐measures ANOVA with Bonferroni's *post hoc*. *N* = 9–10. * *p* < .05 and ** *p* < .01

In addition to quantitative changes in USVs, a common qualitative parameter measured to assess changes in communication is the syllable repertoire (Romano et al., 2013; Scattoni et al., 2011). One of the animals in the uniparental protocol at PD9 presented with 0 calls after reunion and was excluded from the repertoire and spectral parameter analysis because we used a paired analysis to identify the effects of reunion in these parameters. Repertoire analysis of within‐subjects effects at PD9 showed that reunion decreased the proportion of chevron (*F*_1,33_ = 6.87, *p* = .01; Figure 3a) and short calls (*F*_1,33_ = 8.73, *p* < .01; Figure 3g), and increased the proportion of frequency steps in the repertoire (*F*_1,33_ = 6.317, *p* = .01; Figure 3f). A main effect of reunion (*F*_1,33_ = 8.71, *p* < .01) and an interaction between reunion, rearing, and sex (*F*_1,33_ = 8.71, *p* < .01) was identified for composite calls (Figure 3c). Bonferroni *post hoc* analysis revealed that in the biparental rearing protocol, there is an increase in the proportion of composite calls between the before and after reunion recordings only for males (*p* < .01). Additionally, females presented a similar effect in the uniparental rearing protocol (*p* < .01; Figure 3c).

**FIGURE 4:**
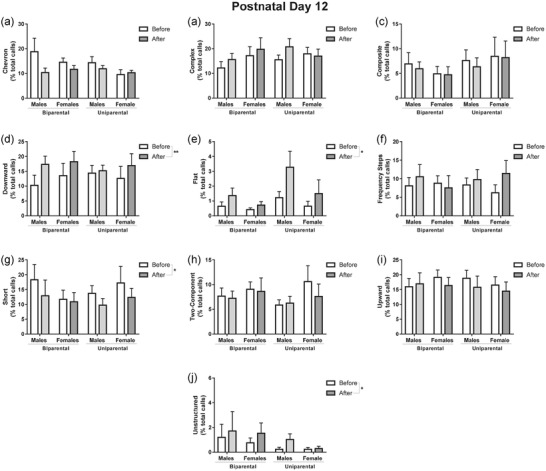
**Reunion alters repertoire at PD12**. Graphs represent the different types of calls performed by the pups during isolation before and after reunion. Each call is represented as the % of the total number of calls in the repertoire. Reunion significantly altered the percentage of calls downward (d), flat (e), short (g), and unstructured (j). The proportion of the call chevron (a), complex (b), composite (c), frequency step (f), two components (h), and upward (i) were not affected by reunion. Data are expressed as mean ± SEM and analyzed with three‐way repeated‐measures ANOVA. *N* = 9–10. * *p* < .05 and ** *p* < .01

At PD12, reunion increased the proportion of downward (*F*_1,33_ = 8.61, *p* < .01; Figure 4d), flat (*F*_1,33_ = 9.74, *p* = .04; Figure 4e), and unstructured (*F*_1,33_ = 7.26, *p* = .01; Figure 4j) calls. Short calls were decreased after reunion (*F*_1,33_ = 4.75, *p* = .03; Figure 4g). No effect of sex or rearing was identified.

### 3.3 Reunion alters vocalization properties mainly at PD9 for both rearing conditions

**FIGURE 5:**
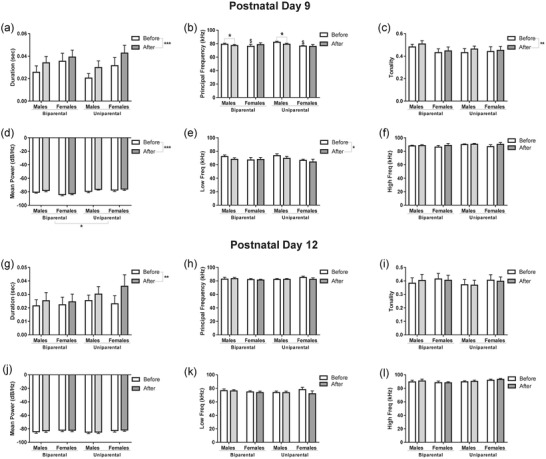
**Vocalization spectral parameters are altered by reunion mainly at PD9**. The spectral parameters duration (a,g), principal frequency (b,h), tonality (c,i), mean power (d,j), low frequency (e,k), and high frequency (f,l) were measured for all the vocalizations performed by the pups in all rearing conditions and sexes at PD9 (a–f) and PD12 (g–l). Reunion increased duration (a), tonality (c), mean power (d), and decreased low frequency (e) at PD9. Also at PD9, reunion decreased principal frequency only in male pups (b). At PD12, reunion increased duration (g). Data are expressed as mean ± SEM and analyzed with three‐way repeated‐measures ANOVA with Bonferroni's *post hoc*. *N* = 9–10. * *p* < .05, ** *p* < .01. $ < .05 versus before males

Quantitative spectral properties of calls were examined to assess if rearing condition could affect the specific properties of the calls. Spectral analysis at PD9 showed that reunion increased duration (*F*_1,33_ = 20.59, *p* < .001; Figure 5a) and tonality (*F*_1,33_ = 7.89, *p* < .01; Figure 5c), but decreased low frequency (*F*_1,33_ = 7.31, *p* = .01; Figure 5e). Within‐subjects interaction between reunion and rearing was identified for principal frequency (*F*_1,33_ = 4.78, *p* = .03; Figure 5b). *Post hoc* analysis revealed that males presented a decrease in principal frequency after the reunion for both rearing conditions (*p* = .03), whereas the females did not present any differences in response to the reunion (*p* > .05). Additionally, females presented lower principal frequencies (*p* = .043) in the first recording (“before”) when compared to males first recordings indicating a baseline sex difference (Figure 5b). Mean power was increased by reunion (within‐subjects, *F*_1,33_ = 18.41, *p* < .001) and affected by rearing condition (between‐subject, *F*_1,33_ = 5.24, *p* = .02). Pups reared by the dam and the sire presented lower mean power than pups reared only by the dam (Figure 5d).

At PD12, the only spectral parameter that was affected by reunion was duration. Similar to PD9, duration increased after the reunion at PD12 (*F*_1,33_ = 7.66, *p* < .01). Rearing conditions and sex had no significant effect on any of the spectral parameters measured at PD12 (Figure 5g).

## 4 DISCUSSION

Maternal potentiation is a behavioral paradigm developed in rats and used to assess filial attachment (Shair, 2014). Although there is considerable literature investigating the specifics of this behavior in rats, not much has been done in mice (Caruso et al., 2020). As mice are the primary species used for the development of genetic disease models, we aimed to identify the effects of sex, rearing conditions, and age on the potentiation of vocalizations in response to reunion with the dam and litter. Although the C57BL/6J strain of mice is not a strain known to partake in biparental care, we decided to use uni‐/biparental rearing condition as a variable based on the study of Brunelli and collaborator that showed that in rats, another species that does not partake in biparental care, rearing conditions influence maternal potentiation (Brunelli et al, 1998). By using two different rearing conditions, males and females, and performing the test at two different ages, we have demonstrated that these factors can influence the outcomes of this test in the C57BL/6J mice strain.

In the present study, we chose two different ages that are commonly used to assess isolation‐induced vocalizations and that have been previously used in the maternal potentiation paradigm, which are PD9 and PD12 (Moles et al., 2004; Young et al., 2010). Here, potentiation of vocalizations occurred only at PD9. At this age, a main effect of reunion was identified, and a *post hoc* analysis of a trend to an interaction between reunion and rearing revealed that the potentiation is mainly observed in the uniparental rearing protocol suggesting that maternal potentiation is influenced by rearing at this age. Our data corroborate previous studies in mice, showing that potentiation in uniparental rearing can occur at PD9 and PD10 in mice (Scattoni et al., 2008; Young et al., 2010). At PD12, no potentiation of vocalizations was identified in our study. Contrary to our findings, potentiation at PD12 has previously been shown in this paradigm in a protocol where there is biparental rearing and an initial isolation of the litter. Moles et al. (2004) demonstrated the potentiation of vocalizations at PD12 in C57BL/6J mice by isolating the litter from the caretakers (dam and sire) for 5 min, then isolating each pup for 3 min, reuniting with the dam for 5 min, and isolating again for 3 min (Moles et al., 2004). The effects of rearing conditions on the performance of the pups might be related to maternal care that was not investigated in the present study. How the sire is influencing maternal potentiation is not known. C57 sires can present “paternal care” behaviors such as retrieval, crouching, and grooming (Stagkourakis et al., 2020), and these behaviors may be attenuating the effects of the isolation during the maternal potentiation paradigm in the biparental rearing condition. The findings that the rearing conditions influence the pups' behavior is in accordance with the literature that shows that rearing conditions have long‐lasting effects on the offspring behavior, including increased susceptibility to alcohol consumption, aggressivity, and anxiogenic‐like behaviors (Ferreyra et al., 2020; Marler et al., 2008; Yohn et al., 2018).

Our findings differ from rat studies where biparental rearing induced potentiation in response to reunion with active or anesthetized dams (Brunelli et al., 1998), but was similar to a study with prairie vole rodents (Robison et al., 2016). In the Brunelli study, the litter was isolated from the dam and or/sire for a 20‐min habituation period. After that, each pup was isolated for 3 min, reunited with a caregiver for 3 min, and isolated again for the final 3 min. In the study developed by Robinson and colleagues, an initial isolation of the whole litter occurred for 15–20 min. The initial isolation of the litter from the caregivers seems to be necessary for potentiation in rats and could be the reason why we did not see a potentiation with our biparental protocol because, in our study, habituation was always done with the dam. But a direct comparison cannot be made between our study and Brunelli et al. (1998) or Robinson et al. (2016) because they used different species. A counterargument is that in the scarce mice literature, the majority of papers that did maternal potentiation in mice did not do the initial isolation of the litter (Scattoni et al., 2008; Young et al., 2010). In the studies of Scattoni et al. (2008) and Young et al. (2010), the pups were isolated and recorded for 5 min, reunited for 5 min, and isolated again for 5 min. No initial isolation of the litter was required. Together, these studies and ours suggest that the maternal potential paradigm might present subtle differences between mice and rats.

An interesting finding in these experiments is that rearing conditions can also change the overall number of vocalizations performed by the pups at both ages the pups were tested at. At PD9, a main effect of rearing showed that pups reared by uniparental rearing call less than biparetal reared pups. At PD12, females in the biparental rearing protocol showed an increase in the number of vocalizations at the “before” recordings when compared to the “before” recordings from males in the same rearing conditions, and the “before” recording of females in uniparental rearing. These findings can have an important impact on developmental research because the isolation‐induced vocalization test is widely used as an early‐life communication/sociability paradigm to evaluate communication alterations during the neonatal period. Moreover, in some experimental paradigms, such as maternal immune activation (Kobayashi et al., 2021; Malkova et al., 2012b), a model of developmental disorders such as autism and schizophrenia (Haddad et al., 2020), and maternal stress models such as limited bedding (Granata et al., 2021; Heun‐Johnson & Levitt, 2016), uniparental rearing is necessary. This type of experimental condition where uniparental rearing is used could have a profound impact on the performance of the pups in the USV test and possibly other tests in adulthood.

The neural substrates of maternal potentiation are not fully elucidated. It is suggested that the potentiation of vocalizations represents reward expectancy and filial attachment because dopaminergic neurotransmission is involved. Previous studies have demonstrated that administration of D2 agonist (quinpirole) inhibits maternal potentiation without altering baseline vocalizations in response to the first isolation (Muller et al., 2005). D1 receptors, on the other hand, seem to be involved only in the isolation‐induced vocalizations but not in the potentiation (Muller et al., 2009). Further studies are needed to fully characterize the networks and neurotransmitter systems involved in maternal potentiation, so this behavior can be more broadly used for the study of attachment/social behavior in neonatal rodents, especially in mice where not many studies have assessed what mechanisms are responsible for potentiation. A common output measurement used to assess communication deficits in developmental studies is the analyses of the syllable repertoire (Romano et al., 2013; Scattoni et al., 2011). To our knowledge, there are no studies that have evaluated this parameter in the maternal potentiation paradigm in rodents. Thus, we did a repertoire analysis for both rearing protocols at the different ages used in our study. Interestingly, we did find alterations in the repertoire in all the ages we assessed potentiation. This suggests that repertoire alteration might be more sensitive to this paradigm than the total number of calls. Although the meaning of the repertoire changes we found in our study is unknown, alteration in the vocal repertoire is a common finding in animal models of developmental disorders in rodents (Binder & Lugo, 2017; Nolan et al., 2020; Romano et al., 2013; Scattoni et al., 2008) and primates (Santana‐Coelho et al., 2021), suggesting that these findings can have significant translatability.

Vocalization spectral parameters can be altered between the first and second isolations in the maternal potentiation paradigm (Young et al., 2010). Thus, we analyzed the average calls duration, principal frequency, tonality, mean power, low frequency, and high frequency. We found that reunion with the dam alters duration, principal frequency, tonality, and low frequency at PD9 independent of rearing conditions, as both protocols induced similar effects in these parameters. Mean power was the only parameter that was influenced by rearing condition and showed a decrease in mean power in biparental rearing when compared to uniparental rearing. Sex had an effect in principal frequency where only males had a decrease in principal frequency in response to reunion. A study by Kaidbey and collaborators has shown that vocalization frequency can be altered between the first to the second isolation in the maternal potentiation paradigm in rats (Kaidbey et al., 2019). However, to our knowledge, this is the first time that a dimorphic effect in principal frequency of USVs has been shown in the maternal potential paradigm. At PD12, there was an effect of reunion only on duration that was increased in the "after" recording. Alteration in the duration of calls has been previously identified in the maternal potentiation paradigm and seems to be a robust and replicable finding (Scattoni et al., 2008).

In summary, our findings suggest that alterations in the maternal potentiation paradigm in C57BL/6J mice can be influenced by rearing conditions, age, and sex. The total number of calls was increased mainly when the offspring were reared by the dam alone. Other outputs, however, were altered in both rearing conditions, suggesting that repertoire and call parameters are more sensitive in this paradigm than the total number of calls. Also, we found more alterations in the repertoire and the spectral properties of the calls at PD9. This age difference might be due to the fact that the peak of USVs in C57BL/6J mice strain in the isolation‐induced paradigm is known to be at PD7, and studies have shown that other insults such as prenatal inflammation (Pendyala et al., 2017) and stressors such as maternal separation (Yin et al., 2016) also cause alterations in vocalizations before postnatal day 12. A more detailed time analysis is necessary to identify the specific time window where the maternal potentiation paradigm can alter vocalizations in C57BL/6J mice and other strains. Also some of the measurements were dependent on sex, such as principal frequency at PD9. It is noteworthy that the fact that the maternal potentiation paradigm was found to be sensitive to environmental factors, such as rearing, brings the opportunity of using this behavioral test to further assess social bonding/attachment in early life models of developmental disorders. Further, in comparison to the regular isolation‐induced vocalization test, the maternal potentiation paradigm has the potential of assessing a learning component of the pup reencounter with the dam and provides a more robust analysis of social and cognitive deficits in early life.

## CONFLICTS OF INTEREST

The authors report no conflicts or potential conflicts of interest.

## STATEMENT OF ETHICS

All mouse work was done according to institutional and IACUC review boards (Baylor University Animal Care and Use Committee). Baylor University maintains an assurance with the Office of Laboratory Animal Welfare ‐ #A3948.

## Data Availability

Data are available on request from the corresponding author.
